# Gene Expression in Hair Follicle Dermal Papilla Cells after Treatment with Stanozolol

**DOI:** 10.4137/bmi.s1173

**Published:** 2008-12-23

**Authors:** M. Reiter, M.W. Pfaffl, M. Schönfelder, H.H.D. Meyer

**Affiliations:** 1 Physiology Weihenstephan Technische Universität München Weihenstephaner Berg 3, D-85354 Freising Germany; 2 Institute of Public Health Research Technische Universität München Conollystraße 32 D-80809 Munich Germany

**Keywords:** anabolic agents, hair follicle dermal papilla cells, gene expression, qRT-PCR, mRNA

## Abstract

Doping with anabolic agents is a topic in sports where strength is crucial, e.g. sprinting, weight lifting and many more. Testosterone and its functional analogs are the drugs of choice taken as pills, creams, tape or injections to increase muscle mass and body performance, and to reduce body fat. Stanozolol (17β-hydroxy-17α-methyl-5α-androst-2-eno[3,2c]pyrazol) is a testosterone analogue with the same anabolic effect like testosterone but its ring structure makes it possible to take it orally. Therefore, stanozolol is one of the most frequently used anabolic steroids.

Common verification methods for anabolic drugs exist, identifying the chemicals in tissues, like hair or blood samples. The idea of this feasibility study was to search for specific gene expression regulations induced by stanozolol to identify the possible influence of the synthetically hormone on different metabolic pathways. Finding biomarkers for anabolic drugs could be supportive of the existing methods and an additional proof for illegal drug abuse.

In two separate cell cultures, human HFDPC (hair follicle dermal papilla cells) from a female and a male donor were treated with stanozolol. In the female cell culture treatment concentrations of 0 nM (control), 1 nM, 10 nM and 100 nM were chosen. Cells were taken 0 h, 6 h, 24 h and 48 h after stimulation and totalRNA was extracted. Learning from the results of the pilot experiment, the male cell culture was treated in 10 nM and 100 nM concentrations and taken after 0 h, 6 h, 24 h and 72 h. Using quantitative real-time RT-PCR expression of characteristics of different target genes were analysed.

Totally 13 genes were selected according to their functionality by screening the actual literature and composed to functional groups: factors of apoptosis regulation were Fas Ligand (FasL), its receptor (FasR), Caspase 8 and Bcl-2. Androgen receptor (AR) and both estrogen receptors (ERα, ERβ) were summarized in the steroid receptor group. The growth factor group included the insulin like growth factor receptor (IGF1R) and growth hormone receptor (GHR). Fibroblast growth factor 2 (FGF2) and keratinocyte growth factor (FGF7) were summarized in the hair cycle factor group. 5α-Steroidreductases (SRD5A1, SRD5A2) represented the enzyme group. Three reference genes were taken for relative quantification: ubiquitin (UBQ), glycerinaldehyde-3-phsophate-dehydrogenase (GAPDH), and β-actin (ACTB).

In cell culture 1 AR, FasR, FGF2 showed significant regulations within one treatment time, significant gene expressions over time were analysed for Caspase 8. In cell culture 2 AR, FasR and SRD5A2 were significantly regulated within one treatment time.

In this feasibility study first biomarker for a screening pattern of anabolic agents could be identified providing the rationality to investigate modified, metabolic pathways in the whole hair follicle.

## Introduction

Anabolic androgenic steroids (AAS) are misused by athletes because of their anabolic properties. Main functions of AAS are stimulation of protein synthesis and an antiglucorticoid effect which increases muscle mass and strength. They influence the central nervous system and increase motivation and performance (Duntas et al. 2003; Hartgens et al. 2004; Hickson et al. 1990; Griggs et al. 1989; Kuhn, 2002). The International Olympic Committee banned the use of synthetic AAS in 1974 by athletes but today doping is still a big topic in almost all kinds of sport. The world anti doping agency (WADA) encourages drug testing laboratories to develop methods to detect AAS like testosterone, 17β-nortestosterone and stanozolol that are the most frequently found steroids in doping samples (Kuhn, 2002; Clasing and Mueller, 2006; Schänzer, 1996). Common used methods in doping analysis are mass spectrometry (MS) based techniques, that directly detect the AAS or their metabolites in different tissues like urine, blood or hair samples. Especially the hair is a very interesting tissue for drug residue analysis because hormones are detectable over a long time and the samples are easily to collect by a non invasive manner (Gleixner et al. 1996; Catlin et al. 1997; Thieme et al. 2000; Ayotte, 2006; Anielskie et al. 2005; Gambelunghe et al. 2007). The intake of AAS influences the organism in many different ways. It can be supposed that these hormonally provoked changes in the metabolism can be seen on the level of mRNA gene expression (Reiter et al. 2007). Taking the hair root to analyse these expressions it is known that androgens act via the dermal papilla cells and influence different kind of growth factors and enzymes (Stenn and Paus, 2001).

The aim of this feasibility study was to investigate regulated target genes in hair follicle cells to identify possible mRNA gene expression regulations aroused by stanozolol. Target genes were selected in functional groups to facilitate the identification of possible biomarker for the different hormones.

## Materials and Methods

### Reagent

The anabolic steroid stanozolol (S, 17β-Hydroxy-17α-methyl-androstano[3,2-c]pyrazole), was received from Sigma-Aldrich (Taufkirchen, Germany) with cell culture grade purity. For treatment the reagents was dissolved in 100% ethanol to a concentration 1mg/ml and diluted with cell culture medium to 1 nM, 10 nM and 100 nM.

### Experiment

Two HFDPC (hair follicle dermal papilla cell) cultures, one from a female (cell culture 1) and one from a male (cell culture 2) donor were treated in different concentrations with the anabolic steroid stanozolol and taken at different treatment time points.

### Cell culture 1

Human HFDPC were ordered from Cell Applications (San Diego, U.S.A). Cells had been cultivated from skin samples of the temple taken during plastic surgery at a 49 year-old, female patient. After establishing a primary cell culture the cells were frozen in serum-free freezing medium and sent cryopreserved (500,000 cells in 1ml). A ready-to-use HFDPC Medium and Supplement Kit (Cell Applications, San Diego, U.S.A) was taken to cultivate the cells, containing Basal Medium, FCS (fetal calf serum), growth factors and antibiotics. For sub cultivation served a kit containing HBSS (HEPES buffered saline solution), trypsin/EDTA solution and neutralizing solution (Cell Applications, San Diego, U.S.A).

The thawed cells were cultivated in collagen coated T-75 flasks (Cell Applications, San Diego, U.S.A) containing 15 ml HFDPC medium at 37 °C in a humidified atmosphere of 5% CO_2_ and sub-cultivated at 85% confluence. Cells were frozen and stored at −80 °C after each splitting that was up to the sixth passage. For the experiment cells from third passage were thawed, given into 12-well plates (ca. 3.4 × 10^4^/well) and cultivated till 85% confluence. Cells were cultured in triplicates with 0 nM (control-treatment), 1 nM, 10 nM and 100 nM stanozolol groups on each plate, sampling took place at 0 h (control-time), 6 h, 24 h and 48 h.

To remove natural containing steroids for all experiments the FCS was stripped by using charcoal, as described by Darbre et al. (1983).

### Cell culture 2

Human HFDPC cells were ordered from Promo Cell (Heidelberg, Germany). Cells had been cultivated from skin samples of the temple taken during plastic surgery at a 40 year-old, male patient. After establishing a primary cell culture the cells were frozen in serum-free freezing medium and sent cryopreserved (500,000 cells in 1ml). A ready-to-use HFDPC Medium and Supplement Kit (Promo Cell, Heidelberg, Germany), containing Basal Medium, FCS (fetal calf serum), basic FGF and Insulin was additionally ordered to cultivate the cells. For sub cultivation served a kit containing HBSS (HEPES buffered saline solution), trypsin/EDTA solution and neutralizing solution (Promo Cell, Heidelberg, Germany).

Cells were cultivated in T-75 flask with 15 ml Medium at 37 °C in a humidified atmosphere of 5% CO_2_ and sub-cultivated at 85% confluence. Collagen coating was not executed because it was not recommended by the supplier. Cells were frozen and stored at −80 °C after each splitting that was up to the sixth passage. For the experiment the cells from third passage were given into 12-well plates (ca. 3.4 × 10^4^/well) and cultivated till 85% confluence. Cells were cultured in triplicates with 0 h (control-treatment), 10 nM and 100 nM stanozolol groups on each plate, sampling took place 0h (control-time), 6 h, 24 h and 72 h.

For treatment, steroids were removed from FCS, as described by Darbre et al. (1983).

### RNA extraction and RNA quality

Total RNA was isolated from HFDPC cells using TriFast (peqLab Biotechnologie GmbH, Erlangen, Germany). The standardized protocol from the supplier was used for the extraction. The principal of this protocol was phenol/chloroform extraction for total RNA. Extracted RNA was dissolved in 10 μl RNAse free water. To quantify the amount of total RNA extracted, optical density (OD) was measured with the photometer (Eppendorf Biophotometer, Hamburg, Germany) for each sample.

RNA integrity and quality control was performed with the Bioanalyzer 2100 (Agilent Technology, Palo Alto, U.S.A). For sample analysis eukaryotic total RNA Nano Assay (Agilent Technology) was taken and the RNA Integrity Number (RIN) served as RNA quality parameter (Fleige et al. 2006, Schroeder et al. 2006). In order to the expected high quality of the total RNA from cell cultures, a set of selected samples from the 6 h treatment were measured as quality references.

### Selection of target genes

Target genes (TG) were selected according to their possible role in the anabolic pathways in the hair follicle. Following TG were chosen as markers by screening the respective literature. Because of the limited total RNA amount, TG were taken for analysis that seemed to be highly influenced by steroids and could show possible gene expression regulations.

Factors of apoptosis regulation were Fas receptor ligand (FasL), Fas receptor (FasR), Caspase 8 and the anti-apototic factor Bcl-2. Androgen receptor (AR) and the estrogen receptors (ERα, ERβ) were summarized in the steroid receptor group. The growth factor group included the insulin like growth factor receptor (IGF1R) and growth hormone receptor (GHR). Fibroblast growth factor 2 (FGF2) and keratinocyte growth factor (FGF7) were summarized in the hair cycle factor group. 5α-Steroidreductases (SRD5A1, SRD5A2) represented the enzyme group.

Ubiquitin (UBQ), glycerinaldehyde-3-phsophate-dehydrogenase (GAPDH) and β-actin (ACTB) were taken as reference genes for relative quantification method.

### Primer design and testing

All primers were designed using published nucleic acid sequences of “Ensembl Genom Browser” (http://www.ensembl.org) and NCBI (www.ncbi.nlm.nih.gov). Primer design and optimization was done with primer design program primer 3 (http://frodo.wi.mit.edu/) with regard to primer dimer formation, self-priming formation and primer annealing temperature at 60 °C. Designed primers were ordered, synthesized and shipped by MWG Biotech (Ebersberg, Germany). Primer testing was performed with a pooled RNA sample of several hair samples, as positive control and RNAse free water as negative control (Qiagen, Hilden, Germany) for each primer set. Generated PCR products were checked for length and primer dimer formation by an agarose gel electrophoresis. All primer sequences, their amplicon size and annealing temperatures are illustrated in Table 1.

### Real-time qRT-PCR

Quantitative real-time RT-PCR was performed at the Rotor Gene 6000 (Corbett Life Science, Sydney, Australia) using SuperScript III Platinum SYBR Green One-Step qPCR Kit (Invitrogen, Carlsbad, U.S.A) by using standard protocol of the supplier. Samples were diluted to 10 ng/μl. For mRNA protection, additionally 50 ng tRNA in 5 μl (Invitrogen, Carlsbad, U.S.A) were added to each sample. 3.8 μl of total RNA solution were taken for one PCR reaction in a total volume of 10 μl. Threshold cycle (Ct) and melting curves were acquired by using the *“quantitation” and “melting curve”* program of the Rotor-Gene 6000 analysis software.

Only genes with clear and single melting peaks were taken for further data analysis. Samples with irregular melting peaks were excluded from the calculation. All samples were baseline corrected and threshold was set manually, using identical threshold levels for one gene in all analyzed samples.

### Data Analysis and Statistics

Data were processed applying relative quantification method comparable to the ΔΔCt-method (2^−ΔΔCt^) (Livak et al. 2001). Expression changes are shown as relative up- or down-regulation normalized by three internal reference genes. For normalization of target gene expression the arithmetic mean (AM) of the following reference genes (RG) were taken: (RG1) GAPDH, (RG2) ACTB and (RG3) UBQ. The mean values served as reference gene index (RGI). RG were excluded when showing significant regulation by treatment or time. This was calculated using the t-test algorithm in Excel (Microsoft, U.S.A). This value was then taken to do normalization and calculate the ΔCt by subtracting the Ct of the RG-Index (RGI) from the Ct of the target gene (TG).

RGI (cell culture 1) = AM (Ct_ACTB_; Ct_GAPDH_; Ct_UBQ_)

RGI (cell culture 2) = AM (Ct_GAPDH_; Ct_UBQ_)

ΔCt = Ct _(RGI)_ − Ct _(TG)_

In a second step the normalized gene expression values were set in relation to the control group (ΔΔCt).

ΔCt _(control/treatment)_ = mean ΔCt value of control treatment

ΔΔCt = ΔCt _(treatment)_ − ΔCt _(control/treatment)_

To show up- and down-regulations of the TG the ratio was calculated provided that DNA was doubled per cycle (2^ΔΔCt^).

Ratio = 2^ΔΔ^_Ct_

Regulations over treatment time were calculated using a 2-Way-Anova with SigmaStat Software. In Excel (Microsoft, U.S.A) ΔΔCt was calculated and statistical analyses were done by using the t-test. All data are illustrated by means ± standard deviation (SD).

## Results

### RNA concentration and RNA integrity

Samples of the cell culture 2 showed a slightly higher mean RNA yield (144.78 ng/μl) compared to the samples of the cell culture 1 (76.9 ng/μl). For the 6 h treatment samples average RIN values were quite similar in cell culture 1 (8.8 ± 0.5) and in cell culture 2 (8.6 ± 2.9).

### Primer testing and PCR

From 19 designed and tested primer pairs three genes did not showed satisfactory PCR results, concerning verifiability, melting curve analysis and in agarose gel electrophoresis. Therefore, FasL, ERα and ERβ were excluded from analysis. All other genes were accepted for further analysis.

### Gene regulation under Stanozolol treatment

Because the reference gene ACTB showed a significant regulation under stanozolol treatment in cell culture 2, only GAPDH and UBQ were taken for RGI calculation.

### Gen expression analysis within one treatment time point (ΔΔCt)

**Figure 1:**
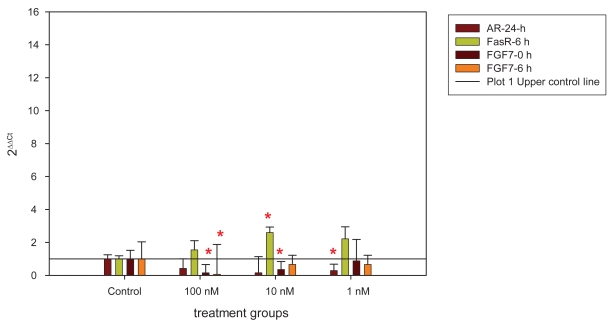
Significant gene expression regulations in cell culture 1. The ratio was calculated by 2^ΔΔCt^ whereby the control was set 1 (upper control line). Data are depicted in bars + standard deviations. Red crosses (*) mark the significantly regulated target genes.

**Figure 2:**
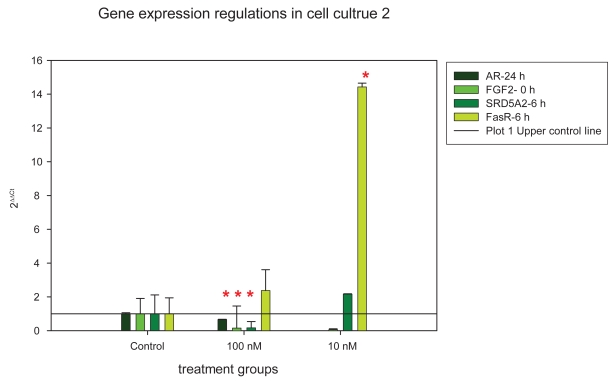
Significant gene expression regulations in cell culture 2. The ratio was calculated by 2^ΔΔCt^ whereby the control was set 1 (upper control line). Data are depicted in bars + standard deviation. Red crosses (*) mark the significantly regulated target genes.

In both cell cultures AR showed a significant down regulation after 24 h and FasR an up- regulation after 6 h of stanozolol treatment. In cell culture 1 the regulation of AR was seen in the 1nM concentration (p = 0.047), in cell culture 2 in the 100 nM concentration (p = 0.014). FasR was up-regulated at 6 h for 10 nM concentration in cell culture 1 (p = 0.023) and cell culture 2 (p = 0.049). Additionally FGF7 was down-regulated at 0 h (100 n M, p = 0.009, 10 nM, p = 0.0d46) and at 6 h (100 nM, p = 0.03) in cell culture 1, in cell culture 2 SRD5A2 (6 h, 100 nM, p = 0.028) and FGF2 (0 h, 100 nM, p = 0.010) showed a significant down-regulation (Figs. 1, 2).

### Gen expression over treatment time (2-Way-Anova)

In the 2-Way-Anova one significant combination of Caspase 8 to treatment concentration (p-value = 0.010) and treatment time (p-value = 0.028) could be calculated in cell culture 1. Caspase 8 in the 1nM concentration was significantly different from all other concentrations and significant differences between 0 h to 6 h (p-value = 0.035) and 0h to 48 h (p-value = 0.029) could be measured for this gene.

## Discussion

In the present study it was tested weather the AAS stanozolol induces specific gene expression regulations in HFDPC. Taking the indicated target genes, first potential genes for an expression pattern could be identified. As feasibility study the experiments were done in cell culture to identify a set of target genes possibly interesting for further analysis in human hair follicle samples.

As expected, all cell culture samples showed a very high RNA quality, a precondition for good qRT-PCR results.

Significant regulations over the time period of treatment were supposed to show trends in gene regulations. The separation in different functional groups could help to identify gene dependent pathways in the hair papilla. Significant influences of stanozolol on gene expression could be shown up to an incubation time of 24 h. After this time the xenobiotic steroid seems to have less influence on gene expressions. This kind of time dependent gene expression regulations were also shown in other studies (Black et al. 1992; Schönfelder and Einspanier, 2003). Most regulations could be seen at 10 nM and 100 nM treatment levels. In both cell cultures AR and FasR showed the same gene expression characteristics under stanozolol treatment at the same time point of treatment. FasR additionally showed comparable regulations at the same hormone concentration. This makes AR and FasR to the first interesting candidate genes for a gene expression pattern of stanozolol. Also a possilbet gender specific difference could be seen because in the male cells the effects of the treatment seemed to induce regulations earlier (6 h) than in the female samples (24 h) (Melchert et al. 1992).

Next to the gender specific effects on gene regulation also the growth phase of the hair follicle could influence the results. A first hind for this assumption could be the different regulations of FGF2 and FGF7 in the untreated samples in both cultures. FGF7 is known to induce the growth phase (anagen phase) in the hair follicle, whereas FGF2 could be taken as its antagonist by inhibiting the morphogenesis (Stenn and Paul, 2001). In cell culture 1 FGF7 was significantly down-regulated what would show that these cells were taken from a hair follicle in the catagen phase. In cell culture 2 FGF2 was significantly down-regulated, indicating the anagen phase. Anyhow the significant regulation of FGF2 was a surprise because several studies identified this factor in the hair follicle but not in the hair papilla (Mitusi et al. 1997; Ota et al. 2002; Schlake et al. 2005).

The significant regulation of FasR and Caspase 8 in cell culture 1 showed first influences on metabolic pathways. The regulation of both factors could indicate a possible influence of stanozolol on the extrinsic way of apoptosis (Reiter et al. 2006).

The significant regulation of IGF1-R is supposed to be an effect of the HFDPC medium because this was already analysed in different studies before (Philpott et al. 1994; Weger et al. 2005; Tang et al. 2003).

## Conclusion

In this feasibility study first steps toward a new screening method via gene expression analysis in hair follicle were explored. A set of 10 genes was tested in cell culture to identify possible candidate genes and physiological influences of stanozolol in the hair papilla. Factors like gender and growth phase of the hair follicle seem to influence gene expression patterns in the cells. Additionally FGF2 known to be expressed in the hair follicle was identified in the hair papilla. AR and FasR can be taken as first candidate genes for a stanozolol treatment pattern because they showed same regulations at the same time point in male and female cell cultures.

Further studies are needed to identify more candidate genes and exclude individual differences in gene expression. Anyhow, in the future the identified substance specific candidate genes could be used for further analysis in hair follicle samples taken from athletes to identify possible treatment with anabolic agents. This could be supportive of the existing test methods in doping analysis and could be an additional proof for illegal drug abuse.

## Figures and Tables

**Table 1:** List of used primer pairs, showing the length of the amplicon (nt), the melt temperature (Tm in °C) and the accession number of the sequence.

Group	Identity		Sequence 5’-3’	Amplicon (nt)	Tm (°C)	Acces. Nr.
Enzymes	SRD5A1	FOR	CTT GAG CCA TTG TGC AGT GT	166	58	ENST00000233239
		REV	GCC TCC CCT TGG TAT TTT GT			
	SRD5A2	FOR	TGA ATA CCC TGA TGG GTG GT	181	60	ENST00000233139
		REV	GGA AAT TGG CTC CAG AAA CAT A			
Growth factors	GHR	FOR	ATC CAC CCA TTG CCC TCA AC	246	60	NM00163
		REV	ATC TCA CAC GCA CTT CAT ATT CC			
	IGF1R	FOR	CAT TTC ACC TCC ACC ACC AC	151	60	NM000875
		REV	AGG CAT CCT GCC CAT CAT AC			
Hair cycle	FGF2	FOR	AGA AGA GCG ACC CTC ACA TC	237	60	M27968
		REV	ACT GCC CAG TTC GTT TCA GT			
	KGF/FGF7	FOR	CCT GAG CGA CAC ACA AGA AG	167	60	M60828
		REV	GCC ACT GTC GCT TCC TTA TT			
Apoptosis factors	FasR	FOR	TTC TGC CAT AAG CCC TGT CC	174	60	NM000043
		REV	CCA CTT CTA AGC CAT GTC CTT C			
	bcl2	FOR	GAG GAT TGT GGC CTT CTT TGA G	170	60	NM000633
		REV	ACA GTT CCA CAA AGG CAT CCC			
	Caspase 8	FOR	TGG CAC TGA TGG ACA GGA G	230	60	NM001228
		REV	GCA GAA AGT CAG CCT CAT CC			
Steroid receptors	AR	FOR	TTG TCC ATC TTG TCG TCT TCG G	237	60	L29496
		REV	TGT CCA GCA CAC ACT ACA CC			
Reference genes	UBQ	FOR	TGA AGA CTC TGA CTG GTA AGA CC	128	60	NM021009
		REV	CAT CCA GCA AAG ATC AGC CTC			
	GAPDH	FOR	GAA GGT GAA GGT CGG AGT CAA	233	60	NM002046
		REV	GCT CCT GGA AGA TGG TGA TG			
	ACTB	FOR	AGTCCTGTGGCATCC ACGAAAC	78	60	NM01101
		REV	GCAGTGATCTCCTTCT GCATCC			
